# Polysome profiling followed by RNA-seq of cardiac differentiation stages in hESCs

**DOI:** 10.1038/sdata.2018.287

**Published:** 2018-12-04

**Authors:** Isabela Tiemy Pereira, Lucia Spangenberg, Anny Waloski Robert, Rocío Amorín, Marco Augusto Stimamiglio, Hugo Naya, Bruno Dallagiovanna

**Affiliations:** 1 Stem Cells Basic Biology Laboratory, Instituto Carlos Chagas - FIOCRUZ-PR, Rua Professor Algacyr Munhoz Mader, 3775, 81.350-010 Curitiba, Brazil; 2 Bioinformatics Unit, Institut Pasteur de Montevideo, Mataojo 2020, CP 11400 Montevideo, Uruguay

**Keywords:** Stem-cell differentiation, RNA sequencing

## Abstract

The regulation of gene expression acts at numerous complementary levels to control and refine protein abundance. The analysis of mRNAs associated with polysomes, called polysome profiling, has been used to investigate the post-transcriptional mechanisms that are involved in different biological processes. Pluripotent stem cells are able to differentiate into a variety of cell lineages, and the cell commitment progression is carefully orchestrated. Genome-wide expression profiling has provided the possibility to investigate transcriptional changes during cardiomyogenesis; however, a more accurate study regarding post-transcriptional regulation is required. In the present work, we isolated and high-throughput sequenced ribosome-free and polysome-bound RNAs from *NKX2-5*^*eGFP/w*^ HES3 undifferentiated pluripotent stem cells at the subsequent differentiation stages of cardiomyogenesis: embryoid body aggregation, mesoderm, cardiac progenitor and cardiomyocyte. The expression of developmental markers was followed by flow cytometry, and quality analyses were performed as technical controls to ensure high quality data. Our dataset provides valuable information about hESC cardiac differentiation and can be used to investigate genes potentially controlled by post-transcriptional mechanisms.

## Background & Summary

Gene expression is controlled by a series of mechanisms, finally leading to protein formation. Continued findings regarding new regulatory mechanisms of gene expression are due to an increased understanding of RNA^1,2^ and more integrative analysis tools^3,4^. The post-transcriptional level of regulation includes transcript synthesis, 5′ capping, splicing, polyadenylation, nuclear export, translation and decay^5^. Translation variants have already been shown as crucial determinants of mammalian gene expression^6,7^, but genome-wide expression profiling is not able to detect the fine adjustment provided by post-transcriptional mechanisms. To overcome this issue, the polysome profiling technique has been used to isolate and further independently analyze ribosome-free and polysome-bound RNAs. RNAs associated with many ribosomes, called polysomes, form large complexes of high molecular weight^8^ and can be easily segregated from ribosome-free RNAs through a sucrose gradient^9–12^.

Protein synthesis control pathways and post-transcriptional mechanisms involved in cell fate commitment are still being established^13–15^. Cardiac tissue formation occurs through precise activation of specific sequential genetic programs to drive cells to differentiation. During embryonic development, cardiomyocytes are derived from the cardiogenic mesoderm through modulation of many pathways and signaling molecules, including bone morphogenetic proteins (BMP), fibroblast growth factors (FGF), NODAL, and canonical and non-canonical Wnt (reviewed by^16^). Moreover, the functional interconnection between transcription factors, their gene targets and signaling pathways delineates cardiomyogenesis and is evolutionarily conserved^17^. The use of isolated human embryonic stem cells (hESC) and induced pluripotent stem cells (iPSC) to derive specific cell lineages *in vitro* raised the possibility of artificially reproducing and studying this differentiation process. Modifications of signaling pathways were used to differentiate hESC to cardiomyocytes^18–21^, which are potential resources for cell therapy and can be used as tools for developmental studies, investigation of endogenous regenerative promotion and cardiac toxicity assays^18,22,23^. However, there is still a lack of detailed information about the complex gene regulatory network that controls cardiac commitment. Unveiling key regulatory elements and molecular signatures of the intermediate differentiation stages can further our current understanding of human cardiac development and produce, select and identify suitable cells for a range of different applications^24^.

**Figure 1:**
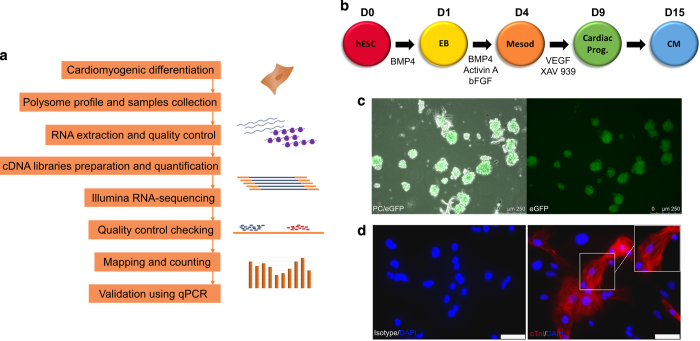
Cardiomyogenic differentiation of hESCs. (**a**) Schematic representation of the steps followed for RNA-seq data generation. (**b**) Schematic representation of the cardiomyogenic differentiation protocol, indicating days of differentiation and timing of specific induction. (**c**) Representative images of EBs during differentiation showing NKX2-5/eGFP expression on D15. Phase contrast (PC) and eGFP fluorescence (left image), eGFP fluorescence (right image). 250 μm scale. (**d**) Representative images of differentiated cardiomyocytes stained for cTnI on D20. Isotype control (left image), cTnI staining (right image). White rectangle as 50 μm scale.

Here, we describe the polysome profiling during the developmental steps of cardiomyogenic commitment. Ribosome-free and polysome-bound mRNAs were isolated and sequenced on D0, D1, D4, D9 and D15, which represents pluripotency, embryoid body (EB) aggregation, cardiac mesoderm, cardiac progenitor and cardiomyocyte stages, respectively (Fig. 1b). Three independent experiments were prepared using 2 to 6 million cells on each time-point mentioned, and technical controls for each analyzed sample and experimental stage were done to ensure high quality data. An overview of the study design is illustrated in Fig. 1a. Our dataset provides valuable information regarding hESC cardiac differentiation and can be used to investigate genes potentially controlled by post-transcriptional mechanisms. Moreover, these data are a powerful tool to explore new elements involved in cardiac cell fate commitment and contributes to the development of novel therapy and research approaches.

## Methods

### Human ESC culture

*NKX2-5*^*eGFP/w*^ HES3 cell lineage was donated by Monash University (Victoria, Australia)^25^. hESCs were cultured on irradiated mouse embryonic fibroblasts (iMEFs) in specific medium composed of Dulbecco’s modified Eagle’s medium (DMEM)/F12 supplemented with 20% KnockOut™ serum replacement (KSR, Gibco™), 100 U/mL penicillin, 100 μg/mL streptomycin, 2 mM L-Glutamine, 1% non-essential amino acid, 55 μM β-mercaptoethanol and 10 ng/mL of human basic fibroblast growth factor (FGF2) (Sigma). They were maintained in a humidified incubator with 5% CO_2_ at 37 °C, with daily medium change and passage every 3–4 days by enzymatic dissociation using 0.05% trypsin/EDTA.

### Cardiomyogenic differentiation of hESCs

A cardiac differentiation protocol was adapted from a previously described source in^18^ and consists of 3 steps: embryoid body (EB) formation, mesoderm induction and cardiac progenitor induction. Initially, 7 × 10^5^ cells/well were plated on Growth Factor Reduced Matrigel Matrix (Corning) 6-well coated dishes and maintained for 72 h in a humidified incubator with 5% CO_2_ at 37 °C. At day 0 (D0) of protocol, hESCs were incubated with collagenase I (1 mg/mL) for 20 min at 37 °C, followed by trypsin-EDTA (0.05%) for approximately 1 min. Immediately after, trypsin was carefully removed, and a medium containing 50% of fetal bovine serum (FBS) and DNAse I (20 U/mL, Invitrogen) was added to the plate. Cells were detached with a cell scraper to avoid single-cell detachment and centrifuged at 230×g for 5 min. After removal of the supernatant, a basal medium composed of StemPro34 (StemPro™-34 SFM, Gibco™), 100 U/mL penicillin, 100 μg/mL streptomycin, 2 mM L-Glutamine, 150 μg/mL transferrin, 50 μg/mL ascorbic acid and 0.45 mM monothioglycerol (MTG) was supplemented with 1 ng/mL BMP4 (R&D systems, cat. 314-BP) and added gently. The cell pellet was resuspended to form small clusters of 10–20 cells, which were seeded into ultra-low attachment 6-well culture plates (Corning® Costar® Ultra-Low Attachment plate) and kept in a humid incubator at 37°C, 5% CO_2_ and 5% O_2_ (hypoxia) for EB aggregation for 24 h. At day 1 (D1), EBs were collected and decanted in a round bottom plastic tube for 30 min. After this period, the supernatant was gently removed, and EBs were resuspended in basal medium supplemented with 10 ng/mL BMP4, 6 ng/mL Activin A (R&D systems, cat. 338-AC) and 5 ng/mL FGF2 (R&D systems, cat. 233-FB) to induce mesoderm specification. After 72 h, on day 4 (D4), the medium was replaced with basal medium supplemented with XAV939 (10 μM/mL) (Tocris, cat. 3748) and VEGF (10 ng/mL) (R&D systems, cat. 293-VE) to induce cells into cardiac progenitors. On days 8 and 11, the medium was replaced with basal medium supplemented with VEGF (10 ng/mL) and BMP4 (1 ng/mL). The cells were kept in a humid incubator at 37 °C, 5% CO_2_ and 5% O_2_ during all the procedure. Three independent differentiation assays were used as experimental replicates. As a control of cardiomyogenic differentiation, hESC were submitted to the same processing without adding any induction factor (non induced differentiation).

### Flow cytometry

EBs were dissociated on D4 using trypsin-EDTA (0.05%) and incubated for 20 min with the surface marker PE-conjugated anti-CD56 (BD cat. 347747, 1:25 in 0.5% PBS/BSA) and 1 μg/μL DAPI. On D9, cells were disaggregated with trypsin-EDTA (0.05%) for 5 min and resuspended in PBS to evaluate eGFP expression. On D15, EBs were disaggregated using 1 mg/mL collagenase I for 16 h and trypsin-EDTA (0.05%) for 5 min, fixed with 4% paraformaldehyde for 20 min and permeabilized with 0.5% Triton X-100 for 30 min. Cells were incubated with anti-troponin T (1:100 in 0.5% PBS/BSA, cardiac isoform Ab-1, Thermo Scientific™, cat. #MS-295-P0) for 30 min followed by Pacific Blue-conjugated anti-mouse (1:1000) for 30 more min. Analyses were carried out using a FACSCanto II flow cytometer (Bd Biosciences) and FlowJo software.

### Immunofluorescence and fluorescent microscopy

EBs on D15 were visualized under a fluorescent microscope and cardiomyogenic committed cells showed NKX2-5/eGFP expression. For immunofluorescence staining, EBs on D15 were disaggregated using 1 mg/mL collagenase I for 16 h and trypsin-EDTA (0.05%) for 5 min and plated on Matrigel coated wells. After 2–5 days, cardiomyocytes were fixed with 4% paraformaldehyde, permeabilized with 0.5% Triton X-100 and blocked with 1% PBS-BSA. Cells were incubated overnight with anti-troponin I (Santa Cruz Biotechnology, cat.: sc-15368, 1:100 in 0.5% PBS/BSA) followed by Alexa 546-conjugated anti-rabbit IgG (1:800, Invitrogen) and 1 μg/μL DAPI. EBs or fixed/stained cardiomyocytes were visualized using a Leica DMI6000B optical microscope, and images were acquired by LAS AF software.

### Polysome profiling

Differentiating cells (2–6 million) at D0, D1, D4, D9 and D15 were treated with 0.1 mg/mL cycloheximide (Sigma-Aldrich) for 10 min at 37 °C, disaggregated with trypsin-EDTA (0.05%) for 10 min and washed twice with 0.1 mg/mL cycloheximide in 1X PBS. Polysome lysis buffer composed of 15 mM Tris HCl pH 7.4, 15 mM MgCl_2_, 300 mM NaCl, 100 μg/mL cycloheximide, 1% Triton X-100, 40 U/μL RNAse Out and 24 U/mL DNAse was used to resuspend cells, followed by 10 min incubation on ice and 10 min centrifugation at 12000×g at 4 °C. Sucrose gradients were prepared with BioComp model 108 Gradient Master using 10% and 50% sucrose solutions (sucrose diluted in polysomal buffer containing 15 mM Tris HCl pH 7.4, 15 mM MgCl_2_ and 300 mM NaCl and prepared in RNAse-free conditions). Clear supernatants from lysed cells were loaded into the 10 to 50% sucrose gradients and centrifuged at 150000×g (SW40 rotor, HIMAC CP80WX HITACHI) for 160 min at 4 °C. Sucrose gradient fractions were separated using ISCO gradient fractionation system (ISCO Model 160 Gradient Former Foxy Jr. Fraction Collector), and the absorbance was monitored at 254 nm to record the polysome profile.

### RNA isolation and quality control

Ribosome-free (fractions 1–3) and polysomal (fractions 10–22) fractions were pooled, and RNA was isolated using the Direct-zol RNA MiniPrep (Zymo Research), following the manufacturer’s instructions. Quality and quantity of RNA were determined using RNA 6000 Pico (for ribosome-free) and Nano (for polysome-bound) kits and an Agilent 2100 Bioanalyzer (Agilent Technologies) and compared to reference samples. Ribosome-free RNA samples ranged from 0.2 to 3.4 μg and polysome-bound from 5 to 16 μg.

### High-Throughput sequencing and data analysis

A total of 30 samples were prepared for sequencing (Table 1). The cDNA libraries were prepared with 200–500 ng of ribosome-free or 2 μg of polysome-bound RNA using the TruSeq Stranded mRNA Sample Preparation kit (Illumina, Inc.). Quality and quantity of cDNA libraries were determined using the DNA 1000 kit, Agilent 2100 Bioanalyzer and KAPA Library Quantification qPCR (KAPA Biosystems). RNA-seq was carried out in an Illumina HiSeq 2500 platform, and raw data quality control was generated using FastQC Reports (https://www.bioinformatics.babraham.ac.uk/projects/fastqc/).

Mapping and counting of sequencing data were performed with Rsubread package^26^ against the new version of the human genome GRCh38. Mapping was done with default parameters and set for unique mapping of the reads. Counting was performed using the annotation of Ensembl (GRCh38). For comparisons of gene expression within and between samples, RPKM values (reads per kilobase per million mapped reads, an expression measure) were determined. For quality check purposes, we performed a PCA analysis, a dimension reduction method of the matrix of counts, to explore associations between variables. Samples of the same condition should cluster together in order to ensure consistency and replicability of results.

### Code availability

R code for data analysis is available upon request. The R version used for this study was 3.3.2. R-packages Rsubread and edgeR were used with versions 1.24.2 and 3.16.5, respectively.

### cDNA preparation and quantitative PCR (qPCR)

Total RNA was extracted using TRIzol® Reagent (Invitrogen), and cDNA synthesis was performed with ImProm-II™ Reverse Transcription System (Promega), following manufacturer’s instructions. Quantitative analysis of transcripts was performed using SYBR® Green (Applied Biosystems) and LightCycler® 96 (Roche) equipment. The primers that were used are shown in Table 2, and for each reaction, 5 pmol of primer and 25 ng of cDNA were used. All samples were evaluated in triplicate.

## Data Records

Flow cytometry data generated during this study were submitted to the FlowRepository (Data Citation 1). FCS files related to each replicate and cardiac differentiation time evaluated (day 3, day 4, day 9 and day 15) are available.

RNA-seq data related to this study were submitted to the NCBI repository SRA (Data Citation 2). Raw RNA-seq data (paired-end fastq files) as generated by Illumina Hiseq 2500 (Data Citation 2). This site serves as a landing page for the study: description of the project, metadata and raw sequencing files can be found there. Individual accession numbers for each biological sample are also provided in Table 1. Counts data and RPKM can be found in file table_genes_counts.xlsx (Data Citation 3). One tab corresponds to the read counts of each sample, and the other, to RPKM values. Each column of each file is labeled with the sample condition, e.g., 1D4P corresponds to biological replicate 1, at day 4 and polysomal RNA fraction.

Beating cardiomyocytes were recorded on video (Online video I, Data Citation 3).

## Technical Validation

### Cardiomyogenic differentiation

**Figure 2:**
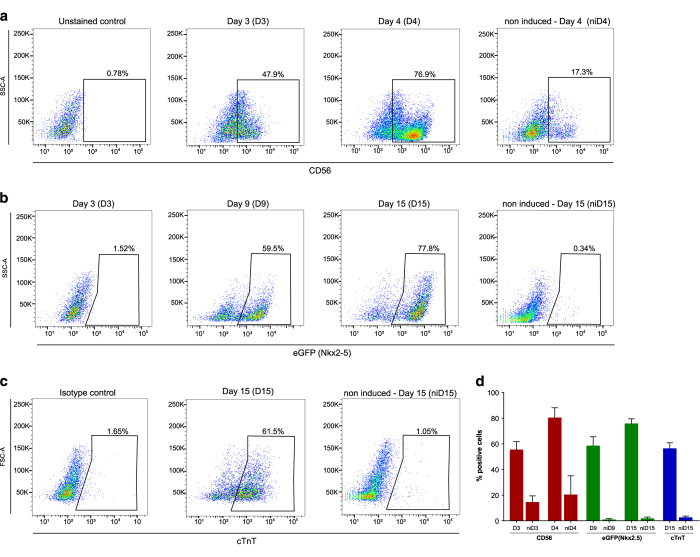
Expression of markers followed during cardiomyogenic differentiation. Flow cytometry analysis of (**a**) D3 and D4 (CD56), (**b**) D3, D9 and D15 (eGFP) and (**c**) D15 (cTnT) differentiating cells. Representative dot plots (n = 3). Non-induced (ni) cells were used as a control for differentiation. (**d**) Quantification of percentage of positive cells for the indicated markers (n = 3) (Data Citation 1).

The *NKX2-5*^*eGFP/w*^ HES3 cell lineage is a reporter human embryonic stem cell (hESC) line that can be used to derive cardiomyocytes and follow the differentiation through eGFP expression^25^. Here, we used a developmentally staged protocol^18,27^ to induce a cardiac mesoderm population on days 3 and 4 and a NKX2-5^+^/cTNT^+^ population by day 15 (Fig. 1b). Beating clusters were observed after 10 days of differentiation (Online video I, Data Citation 3), and cardiomyogenic cells were seen by NKX2-5/eGFP expression (Fig. 1c). Immunostaining using cTNI showed the striations characteristic of sarcomere structures on day 20 of differentiation, as representative control of differentiation protocol (Fig. 1d). To follow the differentiation progress, we established two checkpoints during the cardiomyogenesis protocol: (1) presence of CD56+ cells on days 3–4, which corresponds to mesoderm specification^28^, and (2) NKX2-5/GFP+ cells on day 9, meaning cardiac progenitor commitment. Moreover, cTnT expression was also determined on day 15 and considered proportional to the efficiency of differentiation. Those markers were followed by flow cytometry in all replicate experiments (n = 3) (Fig. 2 and Data Citation 1). Samples used for data acquisition yielded 80.17 ± 7.9% of CD56 + on D4, 58.37 ± 7.1% of eGFP + on D9 and on D15 75.67 ± 3.8% of eGFP^+^ and 56.23 ± 4.5% cTNT^+^ cells (Table 1 and Fig. 2).

### Polysome profiling

**Figure 3:**
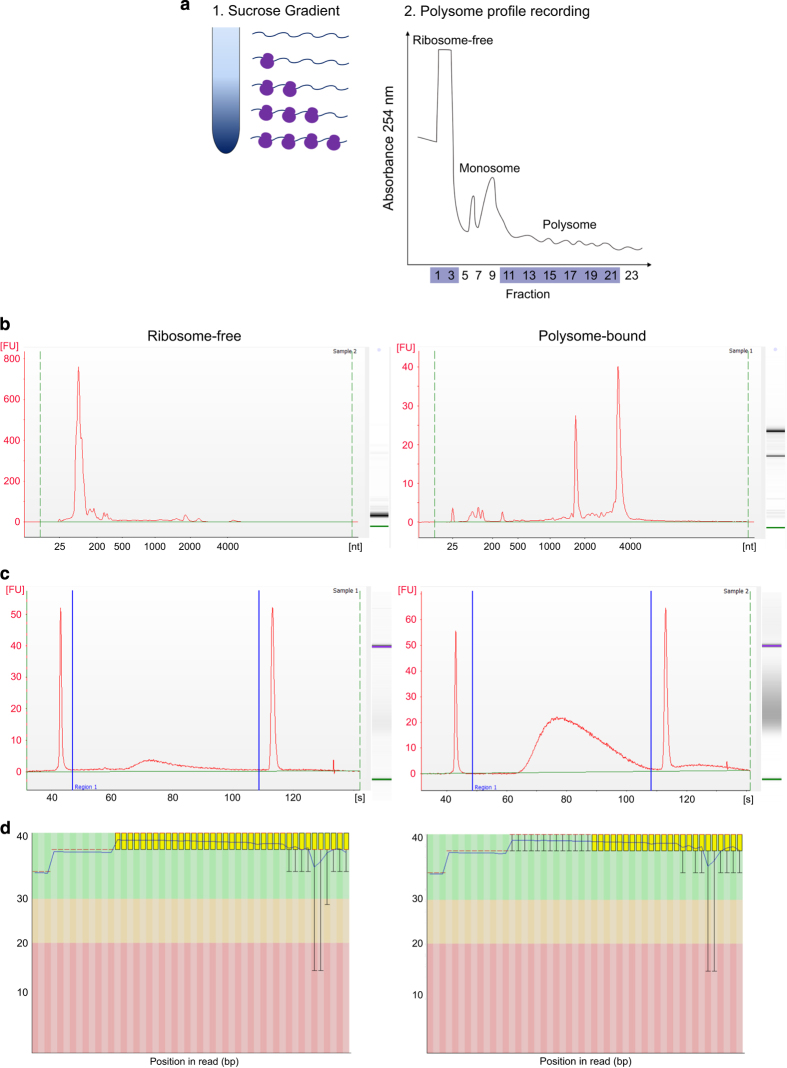
Polysome profiling followed by RNA-seq during cardiomyogenic differentiation. (**a**) Schematic representation of the sucrose gradient used to segregate ribosome-free and ribosome-bound RNAs and representative polysome profile (hESCs - D0 replicate 2) recorded at 254 nm. Ribosome-free and polysome fractions are indicated. (**b**-**d**) Representative quality analysis of ribosome-free and polysome-bound samples. (**a**) RNA quality analysis using Agilent 2100 Bioanalyzer. (**b**) cDNA library quality analysis using Agilent 2100 Bioanalyzer. (**c**) RNA-sequencing reads quality analysis using FastQC. All representative images correspond to D0 hESC sample, replicate 2.

In order to increase the accuracy of the cardiomyogenesis translatome study, we chose to use the polysome profiling methodology to access the polysome-bound RNAs in distinct phases of cardiac differentiation. We performed polysome profiling on D0, D1, D4, D9 and D15 of the differentiation protocol, which represent pluripotency, EB aggregation, cardiac mesoderm, cardiac progenitor and cardiomyocyte stages, respectively (Fig. 1b). Differing densities within the sucrose gradient allowed for the isolation of ribosome-free and polysome-bound RNAs after ultracentrifugation (Fig. 3a). Several fractions were separated using the ISCO gradient fractionation system, while the polysome profiles were recorded using a UV detector (Fig. 3a). The polysome profile derived from each sample was used to determine the fractions that corresponded to ribosome-free (fractions 1–3) and polysome-bound RNAs (fraction 10–22), which were pooled and followed by RNA extraction. One representative image of polysome profile (D0 replicate 2) is shown on Fig. 3a.

### RNA analysis, cDNA libraries and sequencing quality control

Isolated RNAs from ribosome-free and polysome-bound fractions were analyzed for quality and concentration to determine their suitability for RNA-sequencing using an Agilent 2100 BioAnalyzer. Figure 3b shows representative examples of quality results of ribosome-free and polysome-bound samples. Polysome-bound samples showed two distinct picks, which represent 18S and 28S ribosomal RNAs. Those peaks were not shown in ribosome-free samples, which was expected, given the absence of ribosomes. On the other hand, a smaller peak corresponding to tRNAs was observed. All samples measured as high integrity and were considered of high quality to be used on RNA-sequencing.

The cDNA libraries prepared with the TruSeq Stranded mRNA Sample Preparation kit were analyzed to determine quality and quantity using an Agilent 2100 Bioanalyzer (Fig. 3c). As examples, representative images generated by this analysis are shown for ribosome-free and polysome-bound samples. Moreover, cDNA libraries were also quantified by a KAPA Library Quantification kit (data not shown), and these values were used to calculate the sequencing input samples. Raw data derived from sequencing were analyzed using FastQC to determine the quality of the reads by comparing read signals to the probability of accurate base-reading. All samples showed suitable scores, and a ribosome-free and polysome-bound representative analysis is shown (Fig. 3d).

### Biological RNA-seq data

**Figure 4:**
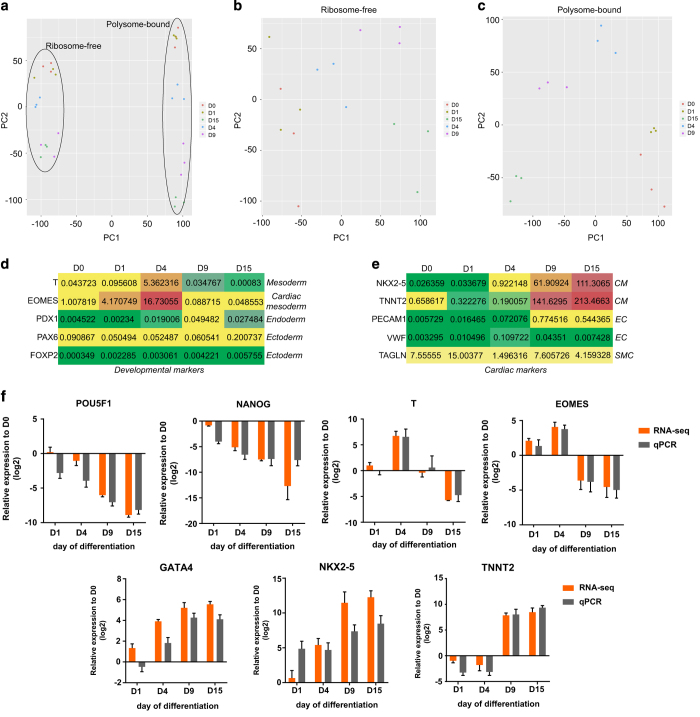
Data quality analysis of RNA-seq and validation. Principal component analysis (PCA) of (**a**) all sequenced samples (total 30 samples), (**b**) ribosome-free and (**c**) polysome-bound samples at D0, D1, D4, D9 and D15 (n = 3). RPKM values (polysome-bound) heatmap of (**d**) developmental markers showing mesoderm, cardiac mesoderm, endoderm and ectoderm genes expression; and (**e**) cardiac markers showing cardiomyocytes (CM), endothelial cells (EC) and smooth muscle cells (SMC) genes expression. (**f**) RNA-seq data validation of cardiac developmental marker gene expression using qPCR. Relative expression to hESC (D0) of pluripotency (POU5F1 and NANOG), mesoderm (T and EOMES) and cardiac markers (GATA4, NKX2-5 and TNNT2) on distinct days of cardiac differentiation using RNA-seq and q-PCR data. Values are expressed in log2 base.

A brief sample description is illustrated in Table 3. Three experimental replicates were done for each differentiation time-point and derived ribosome-free and polysome-bound RNA samples. All sequencing data were deposited at the SRA repository (NCBI) (Data Citation 2). Samples were grouped according to type of RNA fraction (ribosome-free vs. polysome-bound) using principal component analysis (PCA) (Fig. 4a) and according to day of differentiation (D0, D1, D4, D9 and D15) (Fig. 4b, c), indicating the reproducibility of biological replicates. Additionally, RPKM values of developmental markers were plotted on a heatmap (Fig. 4d) to show the specificity of mesoderm commitment among the three germ layers. RPKM values of cardiac markers were also plotted (Fig. 4e) to show the higher cardiomyocyte marker expression when compared to markers of endothelial (EC) and smooth muscle (SMC) cells, other cardiac progenitor derivatives.

### qPCR validation

To identify if our RNA-seq data were compatible with cardiomyogenesis gene expression, we prepared total RNA samples from the same differentiation time-points for qPCR analysis of developmental and cardiac marker expression. Comparing the log2 fold change of hESC on day 0 (D0), we demonstrated the similarity between RNA-seq and qPCR results. POU5F1 (OCT4) and NANOG are transcription factors expressed in pluripotency conditions which compose the pluripotency core regulatory circuitry^29^. These genes represent markers for the pluripotent state and showed a gradual decrease in expression throughout differentiation in our RNA-seq and qPCR results (Fig. 4f). Developmental markers were also analyzed, as the T-box Brachyury/T, which has a conserved role in mesoderm differentiation^30^, and Eomesodermin (EOMES), which expression marks the earliest cardiac mesoderm and promotes formation of cardiovascular progenitors^31^. The mesodermal markers T and EOMES showed increased expression on D4 (mesoderm stage) and were down-regulated on D9 and D15. Finally, the expression of cardiac-related genes such as GATA4, NKX2-5 and TNNT2 was increased during differentiation (Fig. 4f).

## Additional information

**How to cite this article**: Pereira, I. T. *et al*. Polysome profiling followed by RNA-seq of cardiac differentiation stages in hESCs. *Sci. Data*. 5:180287 doi: 10.1038/sdata.2018.287 (2018).

**Publisher’s note**: Springer Nature remains neutral with regard to jurisdictional claims in published maps and institutional affiliations.

## Supplementary Material



## Figures and Tables

**Table 1:** Description of the samples used to generate RNA-sequencing data of distinct days of differentiation.

	Sample	Fraction	Replicate	Deposit
D0	2.23E + 06 cells on day 0	Ribosome-free	Replicate 1	SAMN09405494
		Polysome-bound	Replicate 1	SAMN09405495
	4.02E + 06 cells on day 0	Ribosome-free	Replicate 2	SAMN09405504
		Polysome-bound	Replicate 2	SAMN09405505
	3.18E + 06 cells on day 0	Ribosome-free	Replicate 3	SAMN09405514
		Polysome-bound	Replicate 3	SAMN09405515
D1	4.65E + 05 cells on day 1	Ribosome-free	Replicate 1	SAMN09405496
		Polysome-bound	Replicate 1	SAMN09405497
	2.44E + 06 cells on day 1	Ribosome-free	Replicate 2	SAMN09405506
		Polysome-bound	Replicate 2	SAMN09405507
	2.38E + 06 cells on day 1	Ribosome-free	Replicate 3	SAMN09405516
		Polysome-bound	Replicate 3	SAMN09405517
D4	3.30E + 06 cells on day 4 (76.9% CD56)	Ribosome-free	Replicate 1	SAMN09405498
		Polysome-bound	Replicate 1	SAMN09405499
	4.38E + 06 cells on day 4 (89.2% CD56)	Ribosome-free	Replicate 2	SAMN09405508
		Polysome-bound	Replicate 2	SAMN09405509
	2.16E + 06 cells on day 4 (74.4% CD56)	Ribosome-free	Replicate 3	SAMN09405518
		Polysome-bound	Replicate 3	SAMN09405519
D9	5.04E + 06 cells on day 9 (59.5% eGFP)	Ribosome-free	Replicate 1	SAMN09405500
		Polysome-bound	Replicate 1	SAMN09405501
	6.38E + 06 cells on day 9 (50.7% eGFP)	Ribosome-free	Replicate 2	SAMN09405510
		Polysome-bound	Replicate 2	SAMN09405511
	2.96E + 06 cells on day 9 (64.9% eGFP)	Ribosome-free	Replicate 3	SAMN09405520
		Polysome-bound	Replicate 3	SAMN09405521
D15	4.58E + 06 cells on day 15 (61.5% cTnT/ 77.8% eGFP)	Ribosome-free	Replicate 1	SAMN09405502
		Polysome-bound	Replicate 1	SAMN09405503
	2.56E + 06 cells on day 15 (54% cTnT/ 71.2% eGFP)	Ribosome-free	Replicate 2	SAMN09405512
		Polysome-bound	Replicate 2	SAMN09405513
	3.45E + 06 cells on day 15 (53.2% cTnT/ 78% eGFP)	Ribosome-free	Replicate 3	SAMN09405522
		Polysome-bound	Replicate 3	SAMN09405523
Day of differentiation, number of cells, gradient fraction and replicates information. Total of 30 samples were prepared (Data Citation 2).				

**Table 2:** Primer sequences used for qPCR analysis of cardiac developmental genes.

Gene	Sequence (5’-3’)	Access number	Amplicon (pb)	Reference
*POU5F1*	F: ATGCATTCAAACTGAGGTGCCTGC	NM_001173531	192 pb	(YE *et al.*, 2013)
	R: AACTTCACCTTCCCTCCAACCAGT			
*NANOG*	F: ACCAGAACTGTGTTCTCTTCCACC	NM_024865	200 pb	(ZAEHRES *et al.*, 2005)
	R: CCATTGCTATTCTTCGGCCAGTTG			
*T*	F: AAAGAGATGATGGAGGAACCCGGA	NM_003181	108 pb	(YE *et al.*, 2013)
	R: AGGATGAGGATTTGCAGGTGGACA			
*EOMES*	F: CAAATTCCACCGCCACCAAACTGA	NM_001278182.1	108 pb	(OVCHINNIKOV *et al.*, 2014)
	R: TTGTAGTGGGCAGTGGGATTGAGT			
*GATA4*	F: ACCTGGGACTTGGAGGATAGCAAA	NM_002052	169 pb	(YE *et al.*, 2013)
	R: TCCCATCAGCGTGTAAAGGCATCT			
*NKX2.5*	F: TTAAGTCACCGTCTGTCTCCCTCA	NM_001166175	124 pb	(YE *et al.*, 2013)
	R: ACCGACACGTCTCACTCAGCATTT			
*TNNT2*	F: TGCAGGAGAAGTTCAAGCAGCAGA	NM_000364	155 pb	(YE *et al.*, 2013)
	R: AGCGAGGAGCAGATCTTTGGTGAA			
*TNNI3*	F: GGGGGCCCGGGCTAAGGAGTC	NM_000363.4	183 pb	(SCHITTINI *et al.*, 2010)
	R: AGGGCAGGGGCAGTAGGCAGGAAG			
*GAPDH*	F: GGCGATGCTGGCGCTGAGTAC	NM_002046.3	149 pb	PrimerBlast
	R: TGGTTCACACCCATGACGA			

**Table 3:** Summary of RNA-seq data from ribosome-free and polysome-bound fractions of distinct cardiomyogenic differentiation time-points (n = 3).

	Fraction	Replicate	Processed reads	Mapped reads	% mapped reads	Genes detected
D0	Ribosome-free	Replicate 1	3.40E + 07	2.45E + 07	72.06	17,346
	Polysome-bound	Replicate 1	3.55E + 07	3.02E + 07	84.99	17,690
	Ribosome-free	Replicate 2	3.33E + 07	1.64E + 07	49.40	16,333
	Polysome-bound	Replicate 2	3.47E + 07	2.94E + 07	84.65	16,820
	Ribosome-free	Replicate 3	3.87E + 07	2.78E + 07	71.72	18,019
	Polysome-bound	Replicate 3	3.48E + 07	2.95E + 07	84.93	17,118
D1	Ribosome-free	Replicate 1	3.71E + 07	2.55E + 07	68.57	17,978
	Polysome-bound	Replicate 1	3.73E + 07	3.17E + 07	84.89	17,921
	Ribosome-free	Replicate 2	3.81E + 07	2.55E + 07	66.93	17,207
	Polysome-bound	Replicate 2	3.31E + 07	2.81E + 07	84.82	17,513
	Ribosome-free	Replicate 3	2.52E + 07	1.72E + 07	68.21	17,447
	Polysome-bound	Replicate 3	3.60E + 07	3.11E + 07	86.28	17,907
D4	Ribosome-free	Replicate 1	3.34E + 07	2.89E + 07	86.35	18,057
	Polysome-bound	Replicate 1	3.85E + 07	3.36E + 07	87.17	18,368
	Ribosome-free	Replicate 2	1.39E + 07	8.07E + 06	57.96	16,871
	Polysome-bound	Replicate 2	3.29E + 07	2.83E + 07	85.77	17,824
	Ribosome-free	Replicate 3	2.69E + 07	1.76E + 07	65.33	18,529
	Polysome-bound	Replicate 3	3.37E + 07	2.92E + 07	86.64	18,155
D9	Ribosome-free	Replicate 1	3.55E + 07	2.62E + 07	73.82	18,182
	Polysome-bound	Replicate 1	2.12E + 07	1.90E + 07	89.32	18,552
	Ribosome-free	Replicate 2	2.67E + 07	1.84E + 07	69.02	16,826
	Polysome-bound	Replicate 2	2.65E + 07	2.36E + 07	88.95	17,978
	Ribosome-free	Replicate 3	2.98E + 07	2.16E + 07	72.38	17,678
	Polysome-bound	Replicate 3	2.90E + 07	2.59E + 07	89.38	17,924
D15	Ribosome-free	Replicate 1	2.97E + 07	2.23E + 07	74.96	17,626
	Polysome-bound	Replicate 1	3.25E + 07	2.90E + 07	89.35	18,093
	Ribosome-free	Replicate 2	5.09E + 07	3.61E + 07	70.93	16,636
	Polysome-bound	Replicate 2	5.00E + 07	4.50E + 07	89.98	17,824
	Ribosome-free	Replicate 3	5.22E + 07	3.73E + 07	71.50	15,948
	Polysome-bound	Replicate 3	2.92E + 07	2.60E + 07	89.12	17,580
